# Public health emergency and psychological distress among healthcare workers: a scoping review

**DOI:** 10.1186/s12889-022-13761-1

**Published:** 2022-07-20

**Authors:** Jennifer Palmer, Michael Ku, Hao Wang, Kien Crosse, Alexandria Bennett, Esther Lee, Alexander Simmons, Lauren Duffy, Jessie Montanaro, Khalid Bazaid

**Affiliations:** 1 Co-Primary Investigator, Royal Ottawa Mental Health Centre, Ottawa, ON Canada; 2 Faculty of Medicine, University of Ottawa, Ottawa, ON Canada; 3 Faculty of Medicine, University of Toronto, Toronto, ON Canada; 4 School of Epidemiology and Public Health, Faculty of Medicine, University of Ottawa, Ottawa, ON Canada

**Keywords:** Organizational research, Mental health, Healthcare workers, Pandemics, Scoping review

## Abstract

**Background:**

Pandemics and natural disasters are immensely stressful events for frontline healthcare workers, as they provide patient care to a population undergoing the impacts of the disaster while experiencing such impacts to their personal lives themselves. With increased stressors to an already demanding job, frontline healthcare workers are at a higher risk of adverse effects to their mental health. The current COVID-19 pandemic has already shown to have had significant impact on the mental health of healthcare workers with increased rates of burnout, anxiety and depression. There is already literature showing the utility of individual programs at improving mental health, however, interventions at the organizational level are not well explored. This scoping review aims to provide an overview and determine the utility of a systematic review of the current body of literature assessing the effectiveness of mental health interventions at the organizational level for healthcare workers during or after a public health emergency.

**Methods:**

Electronic databases such as Medline on OVID, CENTRAL, PsycINFO on OVID and Embase on OVID were searched. A targeted search of the grey literature was conducted to identify any non-indexed studies. The population, concept and context approach was used to develop the eligibility criteria. Articles were included if (1) they assessed the impact of interventions to improve wellbeing or reduce the distress on healthcare personnel, first responders or military actively providing medical care; (2) provided quantitative or qualitative data with clearly defined outcomes that focused on established mental health indicators or qualitative descriptions on distress and wellbeing, validated scales and workplace indicators; (3) focused on organizational level interventions that occurred in a public health crisis.

**Results:**

The literature search resulted in 4007 citations and 115 potentially relevant full-text papers. All except 5 were excluded. There were four review articles and one experimental study. There were no other unpublished reports that warranted inclusion.

**Conclusions:**

There is a distinct lack of research examining organizational interventions addressing mental resilience and well-being in healthcare workers in disaster settings. A systematic review in this area would be low yield. There is a clear need for further research in this area.

**Supplementary Information:**

The online version contains supplementary material available at 10.1186/s12889-022-13761-1.

## Introduction

Pandemics, natural disasters, and their aftermath present a time of intense distress for doctors, nurses, and other frontline healthcare workers. Not only do these staff provide care for patients affected by the public health emergency, but they must do so while navigating their own stresses associated with significant changes in their workplace and personal lives. This results in immense physical and psychological pressure that is often beyond what their training and the system they work within is generally expected to support. Consequently, frontline healthcare workers are more likely to experience significant adverse effects on their mental health after working during disasters and pandemics [1–3]. For example, the current COVID-19 pandemic has had a significant impact on the mental health of healthcare workers, including increased rates of burnout, anxiety, depression, and post-traumatic stress disorder (PTSD) [4–6]. The increased stressors in the work environment contribute to physician burnout and are associated with worsened job performance and decreased patient safety, both of which have negative societal implications [7, 8].

There are many ways to support healthcare workers through individual programs and at the organizational level [9]. Organizational level interventions try to address issues at the policy level and thus target the operations of the group (e.g. changing shift patterns, increasing the number of sick days per year). Individual interventions are those that members seek out on their own (e.g. someone who applies mindfulness techniques on their own). Due to the difficulties of instituting large-scale change and more importantly, its assessment, the literature on these mental health interventions is often limited to the individual level and tends to be of lower quality [10]. What evidence does exist suggests that organizational interventions and policies can significantly impact wellbeing and resilience of medical staff, first responders and involved military, while improving patient safety [11]. However, pandemics present unique and challenging circumstances. During the COVID-19 pandemic specifically, despite a lack of outcome-based research, examples of “organizational level” interventions have been implemented and include organization-wide programs for meditation and mindfulness, peer support-networks, managerial debriefs, rapidly accessible mental health professionals, specialized communication training and strategies, and ethical and moral decision supports [12–14].

This scoping review aims to provide an overview and determine the utility of a systematic review of the current body of literature assessing the effectiveness of mental health interventions at the organizational level for healthcare workers who are working during or after a public health emergency.

## Methods

### Scoping review and search strategy

The following scoping review adheres to the recommendations from the Preferred Reporting Items for Systematic Review and Meta-Analysis extension for Scoping Reviews (PRISMA-ScR) statement and follows the methodological framework of a scoping review as outlined by Askey & O’Malley and Levac et al. [15–17].

A peer reviewed search strategy was developed by the research team, which consisted of psychiatrists, psychiatry residents, medical students, and an experienced librarian. Electronic databases searched included Medline on OVID, CENTRAL, PsycINFO on OVID, and Embase on OVID. Search strategies are listed in Additional file 2: Appendix 1. The search did not include specific mental disorders because target literature topics were prevention and control, rather than identification and treatment of specific illnesses. The option to include mental health as a search term was discussed by the research team and was determined to be an appropriate replacement for specific disorders.

To supplement the initial search, a thorough targeted search of the grey literature was conducted to identify any non-indexed studies including unpublished trial data, dissertations/theses, conference proceedings, etc. The reference list of relevant reviews and included studies was also manually searched for relevant studies not captured in the initial search.

### Eligibility criteria

The population, concept, and context (PCC) approach was used to facilitate the development of eligibility criteria and to standardize the screening approach (Table 1). To be included in the review, studies needed to assess the impact of interventions meant to improve wellbeing and resilience and reduce distress among healthcare personnel, first responders, or military actively providing medical care in the context of a public health emergency. Healthcare personnel were defined as physicians, nurses, or allied health professionals providing direct patient care (e.g. respiratory therapists). This was done to highlight the distinct impact of both the mental and physical risks of the pandemic on these individuals, and the unique challenge of replacing these individuals.

**Table 1:** Inclusion and exclusion criteria

	**Population**	**Context**	**Concept**
Included	Healthcare workers (physicians, nursing personnel, allied health professionals active in acute medical teams), military and first responders involved in direct patient care	Healthcare setting (hospitals, community, long-term care) during a public health emergency	Organization level intervention (e.g., communication strategies within teams, changing shift patterns); Measured outcomes for mental health, wellbeing, burnout, sick days, distress, resilience, workplace satisfaction, sick days, and related
Excluded	Allied health care or administration staff not involved in direct patient care, psychologists, social workers, physiotherapists, audiologists, occupational therapists, technicians	Not a healthcare setting, first responders and military during non-healthcare related activities; Setting not during a public health emergency	Individual levels intervention (ex. mindfulness techniques, treatment targeted to individuals with disorder only); Describing risk factors only (no intervention)

Included were peer reviewed articles, experimental studies, and observational studies that described the implementation of a specific intervention. All studies provided either quantitative and/or qualitative data with clearly defined outcomes. Outcomes had to focus on established mental health indicators, and included qualitative descriptions on distress and wellbeing, validated scales, and workplace indicators (such as worker satisfaction and number of sick days taken). Articles must have focused on organizational level interventions that occurred during or shortly after a public health crisis (e.g., epidemic, natural disaster). The search was not limited by language or by year.

Articles were excluded if their populations were not subcategorized by healthcare personnel, first responders, and military. For instance, studies using composite data of first responders and hospital administrative staff without separating these two distinct groups were excluded. Studies that focused on specific mental disorders were included if they met the other criteria.

### Study selection

Inter-rater discrepancies from the initial 100 articles screened were resolved by group discussion and from these conversations the inclusion/exclusion criteria were refined as appropriate. All references identified from the initial search were uploaded and screened using Covidence systematic review software [18]. Pairs of reviewers independently screened each study using a pre-defined criteria form for title and abstract review. A designated independent reviewer resolved any remaining conflicts. Full-text review was then completed, with a designated independent reviewer resolving any discrepancies.

## Results

**Fig. 1:**
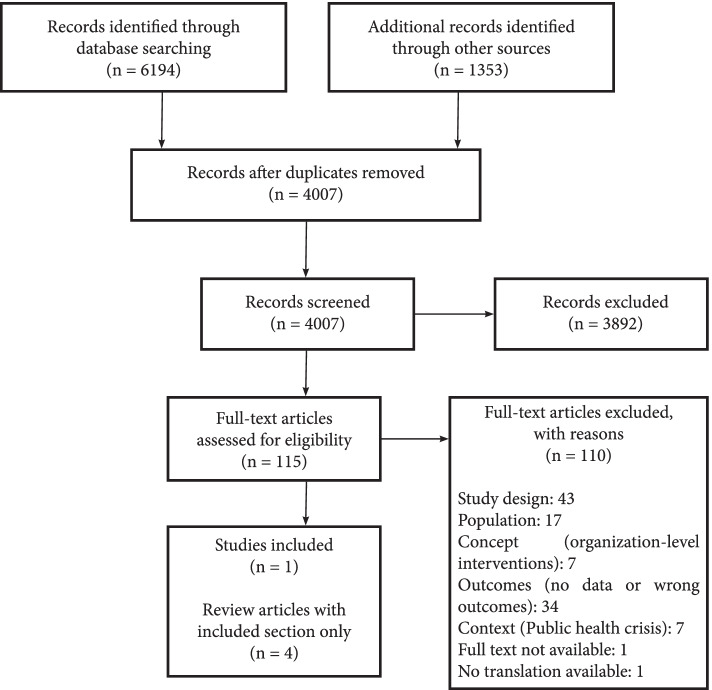
PRISMA flow diagram and list of excluded full-text studies with reasons

The literature search resulted in 4007 citations, of which 115 potentially relevant full-text papers were reviewed (Fig. 1). Only five papers met eligibility criteria. There was one abstract summary with systematic search criteria prepared by the Canadian Agency for Drugs and Technologies in Health (CADTH) that was included. There were no other unpublished reports (grey literature) that warranted inclusion.

The five included articles consisted of four review articles and one experimental study (Table 2). The experimental study by Ke et al. [19] was included in full. Although the review articles each only had one applicable section, given the dearth of data, they were still included. The four review articles include Heath et al. [20], Malcolm et al. [21], Alexander and Klein [22] and Cabarkapa [23].

**Table 2:** Characteristics of included studies

Authors	Country	Year	Study Design	Population	Intervention / Purpose of review
Ke et al. [19]	Taiwan	2017	Cohort study	Physicians and Nurses	“On-site psychological and physical therapy (e.g. debriefing, minilectures, relaxation programs)”
Heath et al. [20]	Australia	2020	Narrative review	Healthcare workers	Summarize the strategies used for increasing resilience in healthcare workers during the COVID-19 pandemic
Malcolm et al. [21]	USA	2005	Review	Emergency services personnel	Examine the literature on the Critical Incident Stress Debriefing (CISD) model of crisis intervention
Alexander and Klein [22]	UK	2009	Review	First responders	Examine the effect of disasters on the psychological and physical welfare and functioning of first responders
Cabarkapa [23]	Australia	2020	Review	Healthcare workers (Nurses and doctors)	Examine the psychological impact of epidemics on healthcare workers, and identify strategies to mitigate psychological distress

The article by Ke et al. was a prospective cohort study conducted in Taiwan after the Tainan City Earthquake in 2016 to assess post-traumatic disorders and resilience in 67 emergency response healthcare workers (35 nurses and 32 physicians) [19]. It describes an intervention that involved on-site psychological and physical therapy available to all and included debriefing, minilectures, as well as relaxation programs by physical therapists, and was in accordance with the guidelines of Psychological First Aid for Provider Care. Administrative chiefs were actively involved and responsible for facilitating conversation around mental health with their teammates and were expected to report any concerns to mental health professionals. Outcomes were measured post-exposure and at 1 month follow-up using the DSM-IV acute stress disorder criteria, with disorder defined as endorsement of any of the assessed symptoms, and resilience defined as resolution of any reported symptom. The incidence of "posttraumatic psychiatric disorders" was 16.4%. At 1 month follow-up there were no symptoms reported by any participants. There was no comparison group.

A narrative review conducted by Heath et al. examined current literature on individual and organizational level strategies that address psychological distress in healthcare workers during the pandemic [20]. Among organizational interventions, staff feedback sessions was identified as a valuable tool to help build personal resilience. These feedback sessions can provide opportunities for staff to participate in decision making about their workplace and help demonstrate the organization’s commitment to engaging and supporting staff, which in turn can contribute to systemic resilience and a sense of organizational justice.

The review by Malcolm et al. evaluated literature on Critical Incident Stress Debriefing (CISD; a specific, formalized seven-phase group discussion associated with a critical incident or traumatic experience.) and other unspecified “debriefing” interventions [21]. Most studies in this review focused on police officers, however there were two studies identified that were relevant. The first study, by Robinson and Mitchell (1993), investigated the utility of debriefing sessions in “hospital and welfare staff and “emergency services personnel” [24]. Both groups reported a significant reduction in their level of distress after the debriefings and rated the debriefing intervention as valuable in contributing to a reduction in stress symptoms.

The review by Alexander and Klein (2009) examined support options for “first responders” in distress [22]. Relevant sections included their review of CISD, Trauma Risk Management (TRiM), and Psychological First Aid (PFA). They concluded that CISD is associated with mostly negative outcomes unless used in the context of a larger evaluative and support program referred to as Critical Incident Stress Management (CISM). TRiM, a method of peer-delivered risk assessment at 3- and 28-days post-event, is relatively new and has mostly been used in military and emergency services. Compared to CISD, there may be lower risk of retraumatization as TRiM does not require detailed descriptions of the event.. The authors remark that the program appears “welcomed by participants”, though more robust evidence is needed. Finally, PFA focuses on a basic hierarchy of needs, starting from basic physical needs and moving towards community support/integration. In contrast to CISD and TRiM, PFA seeks to augment coping strategies and normalize rather than medicalize emotional responses of participants. The authors suggest that some of these qualities may make PFA more acceptable in certain groups such as first responders.

Cabarkapa et al. investigated the psychological impact on health care workers facing epidemics or pandemics [23]. They found that support from supervisors and colleagues was a significant negative predictor for psychiatric symptoms and PTSD. Furthermore, several studies demonstrated that clear communication within the work environment was one of the most important factors for reducing distress amongst healthcare workers. Stigma was identified separately as an important concern, particularly in nursing populations, that could best be supported through administrative and organization-level interventions.

The report provided by Canadian Agency for Drugs and Technologies in Health (CADTH) is a Rapid Response Report based on a limited literature search [25]. Their search was limited to English articles published between 2006 and 2016 examining the clinical effectiveness of integrated peer support programs on treatment of PTSD or operational stress injuries. Despite a search of many databases, no relevant literature was identified.

## Discussion

This scoping review clearly demonstrates a paucity in the literature on organizational interventions to address psychological distress in healthcare workers during public health emergencies and natural disasters. For example, multiple studies have shown that in the face of a pandemic, patient-facing healthcare workers are at risk of increased psychological stress due to a variety of factors such as job stress, personal fear, and lack of support [26, 27]. Furthermore, studies have shown that individual interventions can be helpful in improving mental health [28]. The findings of this current review are in keeping with Pollock et al. [10], a Cochrane review that found a similar lack of evidence surrounding mental health interventions, though this review was limited to epidemic and pandemic settings. Although Pollock et al. [10] had suggested that there may be a greater wealth of evidence in exploring healthcare crises beyond disease outbreaks, the present study shows that there is a lack of evidence across all public health emergencies. While it is difficult to provide practical recommendations at this time given the scarcity of literature concerning this subject, the present scoping review does illustrate two important points, namely that a full systematic review would be fruitless given the quantity of relevant articles, and that further research in this area is critically needed. Given the literature on individual level interventions, there is reason to believe that mental health interventions can be helpful on an organizational level. Specifically, even based on the limited research identified in the present review, there is some evidence to show that debriefing tools may be effective and helpful.

There is a clear need for further research in this area. Primary studies like randomized trials or prospective studies would be ideal to further investigate the benefits of organizational approaches to support the mental health of healthcare workers. Given the inherent difficulties in performing randomized trials with organizations, a practical option would be to examine existing organizational interventions at different institutions and compare the effects they have on healthcare providers’ mental health. Further, it may be easier to start with research in smaller institutions such as family health teams where the focus can still be on healthcare providers, while alleviating the administrative challenges that come with gathering data in larger organizations.

A limitation of the present study is that the quality of the included studies was not assessed. Furthermore, our search did not include articles in non-electronic medical databases, grey literature outside of Canada, and available but non-published data held at organizations themselves. As well, articles that were not in English were excluded.

## Conclusion

There is a prominent gap in research examining organizational interventions to improve mental resilience and well-being in healthcare personnel in natural disasters and public health emergency settings. This scoping review shows both that a systematic review in this area at this time would be low yield, and that there is a clear need for further research in this area. Future research is vital to protecting the safety and well-being of healthcare workers and for maintaining the successful provision of quality healthcare. By recognizing the clear evidence of a vacuum of literature on organizational supports for clinicians’ mental health, and the significant need for this, it is hoped that clinicians and researchers will be encouraged to explore and investigate this critical yet understudied area of research.

## Supplementary Information

**Additional file 1.**

**Additional file 2: Appendix 1.** Search Strategy for Scoping Review.

## Data Availability

Not applicable.
