# Supplementary material for: Public health emergency and psychological distress among healthcare workers: a scoping review
Source: BMC Public Health. 2022 Jul 20;22:1396. doi: 10.1186/s12889-022-13761-1 (PMC9299961; doi:10.1186/s12889-022-13761-1)
Supplement: Supplementary file 2 — Additional file 2: Appendix 1. Search Strategy for Scoping Review. [file 12889_2022_13761_MOESM2_ESM.pdf]

Appendix 1: Search Strategy for Scoping Review

MEDLINE(R) ALL (OVID, 1946 to June 24, 2020)
1. exp emergency responders/ or health personnel/ or emergency medical dispatcher/ or exp medical staff/ or exp nurses/ or exp nursing staff/ or exp medical staff, hospital/ or nursing staff, hospital/ or exp physicians/ or Health workforce/
2. Military Personnel/
3. ((militar* or armed force*) adj2 (personnel* or person* or men or man or woman or women or
people or group*or troop*)).ti,ab,kf.
4. soldier*.ti,ab,kf.
5. (paramedic* or para-medic? or firefighter* or fire fighter* or policem?n or police officer*).ti,ab,kf.
6. ((ambulance* or rescue*) adj2 personnel*).ti,ab,kf.
7. ((medical or first* or disaster*) adj1 responder*).ti,ab,kf.
8. (general practitioner* or clinician* or physician* or doctor*).ti,ab,kf.
9. ((medical* or clinical* or healthcare* or health care* or nursing) adj1 (staff* or personnel* or
professional* or practitioner* or worker* or workforce*)).ti,ab,kf.
10. (nurse* or respiratory therapist*).ti,ab,kf.
11. (emergenc* adj2 dispatcher*).ti,ab,kf.
12. (surgeon* or psychiatrist* or radiologist* or obstetrician* or gyn?ecologist* or an?esthesiologist* or dermatologist* or oncologist* or rheumatologist* or neurologist* or pathologist* or p?ediatrician* or cardiologist* or urologist* or geriatrician* or gerontologist*).ti,ab,kf.
13. or/1-12
14. mental health/
15. mental health.ti,ab,kf.
16. psychological distress/
17. exp Stress, Psychological/pc [Prevention & Control]
18. occupational stress/pc or burnout, professional/pc
19. ((moral* or emotional*) adj2 (injur* or distress* or disturb*) adj3 (prevent* or reduc* or decreas* or diminish* or diminu* or lessen*)).ti,ab,kf.
20. (stress* adj3 (prevent* or reduc* or decreas* or diminish* or diminu* or lessen*)).ti,ab,kf.
21. (psychological adj1 (injur* or distress* or disturb*) adj3 (prevent* or reduc* or decreas* or
diminish* or diminu* or lessen*)).ti,ab,kf.
22. (burnout* adj3 (prevent* or reduc* or decreas* or diminish* or diminu* or lessen*)).ti,ab,kf.
23. Resilience, Psychological/
24. post traumatic growth*.ti,ab,kf.
25. (resilien* or well-being* or wellbeing* or wellness*).ti,ab,kf.
26. Job Satisfaction/
27. ((employment* or job* or workplace*) adj2 satisf*).ti,ab,kf.
28. Sick Leave/

29. (sick* adj2 (leave* or day*)).ti,ab,kf.
30. or/14-29
31. disease outbreaks/ or epidemics/ or pandemics/
32. (pandemic* or epidemic*).ti,ab,kf.
33. ((health or virus* or disease*) adj3 outbreak*).ti,ab,kf.
34. (public health adj3 (emergenc* or trauma* or crisis*)).ti,ab,kf.
35. disasters/ or disaster planning/ or exp natural disasters/ or tsunamis/ or volcanic eruptions/ or relief
work/ or rescue work/
36. disaster*.ti,ab,kf.
37. ((relief or aid* or rescue* or humanitarian*) adj2 (work* or effort*)).ti,ab,kf.
38. (flood* or tsunami* or earthquake* or landslide* or cyclon* or hurricane* or tornado* or
avalanche* or wildfire* or (volcan* adj2 erupt*)).ti,ab,kf.
39. terrorism/ or bioterrorism/ or mass casualty incidents/ or september 11 terrorist attacks/
40. Accidents, Aviation/
41. ((hostage* adj2 (crisis* or situation*)) or kidnapping* or ((plane* or air) adj2 crash*) or mass
shooting* or terror* attack* or terrorism* or bioterrorism* or mass casualt* or bombing*).ti,ab,kf.
42. coronavirus/ or middle east respiratory syndrome coronavirus/ or sars virus/ or Coronavirus
Infections/ or Severe Acute Respiratory Syndrome/
43. Influenza A Virus, H1N1 Subtype/
44. Hemorrhagic Fever, Ebola/ or Ebolavirus/
45. (SARS or MERS).ti,ab,kf.
46. (SARSCOV* or Severe Acute Respiratory Syndrome* or sudden acute respiratory syndrome* or
MERSCoV* or Middle East Respiratory or camel flu or EMC 2012).ti,ab,kf.
47. (H1N1* or Ebola* or swine flu*).ti,ab,kf.
48. (nCoV* or 2019nCoV or 19nCoV or COVID19* or COVID-19* or COVID or SARS-COV-2 or SARSCOV-2
or SARSCOV2 or Coronavirus* or corona virus* or betacoronavirus*).ti,ab,kf,nm,ox,rx,px.
49. ((Wuhan or Hubei) adj5 pneumonia).ti,ab,kf.
50. or/31-49
51. 13 and 30 and 50
Results: 1,903 references retrieved

Embase (OVID, 1947 to 2020 June 24)
1. health care personnel/ or nursing staff/ or rescue personnel/ or respiratory therapist/
2. emergency medical dispatcher/
3. medical staff/
4. exp nurse/
5. exp physician/

6. health workforce/
7. military personnel/
8. ((militar* or armed force*) adj2 (personnel* or person* or men or man or woman or women or
people or group*or troop*)).ti,ab,kw.
9. soldier*.ti,ab,kw.
10. (paramedic* or para-medic? or firefighter* or fire fighter* or policem?n or police officer*).ti,ab,kw.
11. ((ambulance* or rescue*) adj2 personnel*).ti,ab,kw.
12. ((medical or first* or disaster*) adj1 responder*).ti,ab,kw.
13. (general practitioner* or clinician* or physician* or doctor*).ti,ab,kw.
14. ((medical* or clinical* or healthcare* or health care* or nursing) adj1 (staff* or personnel* or
professional* or practitioner* or worker* or workforce*)).ti,ab,kw.
15. (nurse* or respiratory therapist*).ti,ab,kw.
16. (emergenc* adj2 dispatcher*).ti,ab,kw.
17. (surgeon* or psychiatrist* or radiologist* or obstetrician* or gyn?ecologist* or an?esthesiologist* or dermatologist* or oncologist* or rheumatologist* or neurologist* or pathologist* or p?ediatrician* or
cardiologist* or urologist* or geriatrician* or gerontologist*).ti,ab,kw.
18. or/1-17
19. mental health/ or psychological well-being/
20. mental health.ti,ab,kw.
21. distress syndrome/pc [Prevention]
22. exp stress/pc [Prevention]
23. exp burnout/pc [Prevention]
24. ((moral* or emotional*) adj2 (injur* or distress* or disturb*) adj3 (prevent* or reduc* or decreas* or
diminish* or diminu* or lessen*)).ti,ab,kw.
25. (stress* adj3 (prevent* or reduc* or decreas* or diminish* or diminu* or lessen*)).ti,ab,kw.
26. (psychological adj1 (injur* or distress* or disturb*) adj3 (prevent* or reduc* or decreas* or
diminish* or diminu* or lessen*)).ti,ab,kw.
27. (burnout* adj3 (prevent* or reduc* or decreas* or diminish* or diminu* or lessen*)).ti,ab,kw.
28. psychological resilience/
29. post traumatic growth*.ti,ab,kw.
30. (resilien* or well-being* or wellbeing* or wellness*).ti,ab,kw.
31. job satisfaction/
32. ((employment* or job* or workplace*) adj2 satisf*).ti,ab,kw.
33. medical leave/
34. (sick* adj2 (leave* or day*)).ti,ab,kw.
35. or/19-34
36. (public health adj3 (emergenc* or trauma* or crisis*)).ti,ab,kw.
37. epidemic/ or pandemic/ or pandemic influenza/

38. (pandemic* or epidemic*).ti,ab,kw.
39. ((health or virus* or disease*) adj3 outbreak*).ti,ab,kw.
40. exp coronavirinae/
41. exp "influenza a virus (h1n1)"/
42. exp Coronavirus infection/
43. Ebola hemorrhagic fever/
44. (coronavirus* or corona virus* or betacoronavirus*).ti,ab,kw.
45. (nCoV* or 2019nCoV or 19nCoV or COVID19* or COVID-19* or COVID or SARS-COV-2 or SARSCOV-2
or SARSCOV2 or Severe Acute Respiratory Syndrome Coronavirus 2 or Severe Acute Respiratory
Syndrome Corona Virus 2).ti,ab,kw.
46. ((Wuhan or Hubei) adj5 pneumonia).ti,ab,kw.
47. (SARS or MERS).ti,ab,kw.
48. (SARSCOV* or Severe Acute Respiratory Syndrome* or sudden acute respiratory syndrome* or
MERSCoV* or Middle East Respiratory or camel flu or EMC 2012).ti,ab,kw.
49. (H1N1* or Ebola* or swine flu*).ti,ab,kw.
50. exp disaster/
51. disaster planning/
52. avalanche/ or earthquake/ or landslide/ or storm surge/ or tsunami/ or volcano/
53. flooding/
54. hurricane/ or tornado/
55. wildfire/ or forest fire/
56. disaster*.ti,ab,kw.
57. ((relief or aid* or rescue* or humanitarian*) adj2 (work* or effort*)).ti,ab,kw.
58. (flood* or tsunami* or earthquake* or landslide* or cyclon* or hurricane* or tornado* or
avalanche* or wildfire* or (volcan* adj2 erupt*)).ti,ab,kw.
59. exp terrorism/
60. aircraft accident/
61. hostage/
62. kidnapping/
63. ((hostage* adj2 (crisis* or situation*)) or kidnapping* or ((plane* or air) adj2 crash*) or mass
shooting* or terror* attack* or terrorism* or bioterrorism* or mass casualt* or bombing*).ti,ab,kw.
64. or/36-63
65. 18 and 35 and 64
66. conference abstract.pt.
67. 65 not 66
Results: 2,336 references retrieved

CINAHL (EBSCOHost)

#	Query	Results
S1	(MH "Health Personnel") OR (MH "Emergency Medical Technicians") OR (MH "Respiratory Therapists") OR (MH "Expert Clinicians+") OR (MH "Medical Staff+") OR (MH "Nurses+") OR (MH "Physicians+") OR (MH "Rapid Response Team") OR (MH "Military Personnel+") OR (MH "Police")	425,630
S2	((militar* or armed force*) N2 (personnel* or person* or men or man or woman or women or people or group*or troop*))	18,022
S3	soldier*	13,642
S4	(paramedic* or "para-medic?" or firefighter* or "fire fighter*" or policem?n or "police officer*")	25,119
S5	((ambulance* or rescue*) N2 personnel*)	1,692
S6	((medical or first* or disaster*) N1 responder*)	5,420
S7	("general practitioner*" or clinician* or physician* or doctor*)	409,677
S8	((medical* or clinical* or healthcare* or "health care*" or nursing) N1 (staff* or personnel* or professional* or practitioner* or worker* or workforce*))	537,273
S9	(nurse* or "respiratory therapist*")	528,199
S10	(emergenc* N2 dispatcher*)	110
S11	(surgeon* or psychiatrist* or radiologist* or obstetrician* or gyn?ecologist* or an?esthesiologist* or dermatologist* or oncologist* or rheumatologist* or neurologist* or pathologist* or p?ediatrician* or cardiologist* or urologist* or geriatrician* or gerontologist*)	143,923
S12	S1 OR S2 OR S3 OR S4 OR S5 OR S6 OR S7 OR S8 OR S9 OR S10 OR S11	1,350,168
S13	(MH "Mental Health")	38,749
S14	mental health	156,186

S15	(MH "Stress+/PC")	11,631
S16	((moral* or emotional*) N2 (injur* or distress* or disturb*) N3 (prevent* or reduc* or decreas* or diminish* or diminu* or lessen*))	4,901
S17	(stress* N3 (prevent* or reduc* or decreas* or diminish* or diminu* or lessen*))	24,889
S18	(psychological N1 (injur* or distress* or disturb*) N3 (prevent* or reduc* or decreas* or diminish* or diminu* or lessen*))	1,051
S19	(burnout* N3 (prevent* or reduc* or decreas* or diminish* or diminu* or lessen*))	3,855
S20	(resilien* or well-being* or wellbeing* or wellness*)	116,406
S21	"post traumatic growth*"	361
S22	(MH "Job Satisfaction")	22,287
S23	((employment* or job* or workplace*) N2 satisf*)	30,354
S24	(MH "Sick Leave")	5,388
S25	(sick* N2 (leave* or day*))	6,924
S26	S13 OR S14 OR S15 OR S16 OR S17 OR S18 OR S19 OR S20 OR S21 OR S22 OR S23 OR S24 OR S25	316,490
S27	(MH "Disease Outbreaks")	31,154
S28	pandemic* or epidemic* or ((health or virus* or disease*) N3 outbreak*)	60,595
S29	("public health" N3 (emergenc* or trauma* or crisis*))	2,468

S30	(MH "Coronavirus+")	1,042
S31	(MH "Coronavirus Infections+")	4,340
S32	(MH "Influenza, Pandemic (H1N1) 2009")	2,168
S33	(MH "Influenza A Virus, H1N1 Subtype")	3,412
S34	(MH "Ebola Virus")	1,281
S35	(MH "Hemorrhagic Fever, Ebola") OR (MH "Hemorrhagic Fever with Renal Syndrome") OR (MH "Dengue Hemorrhagic Fever")	4,318
S36	SARS or MERS	4,065
S37	SARSCOV* or "Severe Acute Respiratory Syndrome*" or "sudden acute respiratory syndrome*" or MERSCoV* or "Middle East Respiratory" or "camel flu" or "EMC 2012"	3,601
S38	H1N1* or Ebola* or "swine flu*"	12,290
S39	nCoV* or 2019nCoV* or 19nCoV* or COVID19* or "COVID-19*" or COVID or SARS-COV-2 or SARSCOV-2 or SARSCOV2 or Coronavirus* or "corona virus*" or betacoronavirus*	8,726
S40	(Wuhan or Hubei) N5 pneumonia*	48
S41	(MH "Disasters+")	34,780
S42	disaster*	32,994
S43	(MH "Rescue Work+")	3,698

S44	(MH "Humanitarian Aid")	3,749
S45	((relief or aid* or rescue* or humanitarian*) N2 (work* or effort*))	28,499
S46	(flood* or tsunami* or earthquake* or landslide* or cyclon* or hurricane* or tornado* or avalanche* or wildfire* or (volcan* N2 erupt*))	14,180
S47	(MH "Terrorism+")	7,247
S48	(MH "Accidents, Aviation")	825
S49	(MH "Hostages")	42
S50	(MH "Kidnapping")	327
S51	((hostage* N2 (crisis* or situation*)) or kidnapping* or ((plane* or air) N2 crash*) or "mass shooting*" or "terror* attack*" or terrorism* or bioterrorism* or "mass casualt*" or bombing*)	12,197
S52	S27 OR S28 OR S29 OR S30 OR S31 OR S32 OR S33 OR S34 OR S35 OR S36 OR S37 OR S38 OR S39 OR S40 OR S41 OR S42 OR S43 OR S44 OR S45 OR S46 OR S47 OR S48 OR S49 OR S50 OR S51	144,411
S53	S12 AND S26 AND S52	3,177
S54	S12 AND S26 AND S52<br>Peer Reviewed; Research Article	607

Results: **607 references** retrieved

APA PsycInfo (OVID, 1806 to June Week 4 2020)
1. health personnel/ or exp medical personnel/ or rescue workers/
2. clinicians/
3. exp military personnel/
4. fire fighters/ or first responders/ or paramedics/ or police personnel/ or rescue workers/

5. ((militar* or armed force*) adj2 (personnel* or person* or men or man or woman or women or
people or group*or troop*)).tw.
6. soldier*.tw.
7. (paramedic* or para-medic? or firefighter* or fire fighter* or policem?n or police officer*).tw.
8. ((ambulance* or rescue*) adj2 personnel*).tw.
9. ((medical or first* or disaster*) adj1 responder*).tw.
10. (general practitioner* or clinician* or physician* or doctor*).tw.
11. ((medical* or clinical* or healthcare* or health care* or nursing) adj1 (staff* or personnel* or
professional* or practitioner* or worker* or workforce*)).tw.
12. (nurse* or respiratory therapist*).tw.
13. (emergenc* adj2 dispatcher*).tw.
14. (surgeon* or psychiatrist* or radiologist* or obstetrician* or gyn?ecologist* or an?esthesiologist* or
dermatologist* or oncologist* or rheumatologist* or neurologist* or pathologist* or p?ediatrician* or
cardiologist* or urologist* or geriatrician* or gerontologist*).tw.
15. or/1-14
16. mental health/ or exp well being/
17. mental health.tw.
18. distress/
19. exp stress/
20. occupational health psychology/
21. ((moral* or emotional*) adj2 (injur* or distress* or disturb*) adj3 (prevent* or reduc* or decreas* or diminish* or diminu* or lessen*)).tw.
22. (stress* adj3 (prevent* or reduc* or decreas* or diminish* or diminu* or lessen*)).tw.
23. (psychological adj1 (injur* or distress* or disturb*) adj3 (prevent* or reduc* or decreas* or
diminish* or diminu* or lessen*)).tw.
24. (burnout* adj3 (prevent* or reduc* or decreas* or diminish* or diminu* or lessen*)).tw.
25. "resilience (psychological)"/ or posttraumatic growth/
26. post traumatic growth*.tw.
27. (resilien* or well-being* or wellbeing* or wellness*).tw.
28. job satisfaction/
29. ((employment* or job* or workplace*) adj2 satisf*).tw.
30. employee leave benefits/
31. (sick* adj2 (leave* or day*)).tw.
32. or/16-31
33. epidemics/ or pandemics/
34. (pandemic* or epidemic*).tw.
35. ((health or virus* or disease*) adj3 outbreak*).tw.
36. (public health adj3 (emergenc* or trauma* or crisis*)).tw.
37. organizational crises/

38. exp Disasters/
39. emergency preparedness/
40. disaster*.tw.
41. ((relief or aid* or rescue* or humanitarian*) adj2 (work* or effort*)).tw.
42. (flood* or tsunami* or earthquake* or landslide* or cyclon* or hurricane* or tornado* or
avalanche* or wildfire* or (volcan* adj2 erupt*)).tw.
43. exp terrorism/ or hostages/
44. kidnapping/
45. air traffic accidents/
46. ((hostage* adj2 (crisis* or situation*)) or kidnapping* or ((plane* or air) adj2 crash*) or mass
shooting* or terror* attack* or terrorism* or bioterrorism* or mass casualt* or bombing*).tw.
47. swine influenza/
48. (SARS or MERS).tw.
49. (SARSCOV* or Severe Acute Respiratory Syndrome* or sudden acute respiratory syndrome* or MERSCoV* or Middle East Respiratory or camel flu or EMC 2012).tw.
50. (H1N1* or Ebola* or swine flu*).tw.
51. (nCoV* or 2019nCoV or 19nCoV or COVID19* or COVID-19* or COVID or SARS-COV-2 or SARSCOV-2 or SARSCOV2 or Coronavirus* or corona virus* or betacoronavirus*).tw.
52. ((Wuhan or Hubei) adj5 pneumonia).tw.
53. or/33-52
54. 15 and 32 and 53
55. (book or authored book or edited book or dissertation abstract).pt.
56. 54 not 55
Results: 1,348 references retrieved
